# Ectodermal dysplasia – Maxillary and mandibular alveolar reconstruction with dental rehabilitation: A case report and review of the literature

**DOI:** 10.4103/0970-0358.63969

**Published:** 2010

**Authors:** Sanjeev N. Deshpande, Vikas Kumar

**Affiliations:** Department of Plastic Surgery, Gokuldas Tejpal Hospital, Mumbai, India

**Keywords:** Alveolar reconstruction, dental implants, ectodermal dysplasia

## Abstract

Ectodermal dysplasia is a rare group of inherited disorders characterized by aplasia or dysplasia of tissues of ectodermal origin, such as hair, nails, teeth and skin. Dental manifestations include hypodontia, complete anodontia or malformed teeth. Oral rehabilitation is the major surgical challenge in such patients. It frequently requires alveolar reconstruction followed by dental implants. We report a case of hypohidrotic ectodermal dysplasia, which was managed with reconstruction of both the upper and the lower alveolus using free fibula flaps with dental rehabilitation using osseointegrated implants.

## INTRODUCTION

Ectodermal dysplasia patients require both functional and aesthetic corrections of the face. The absence of alveolar bone and teeth is a difficult reconstructive challenge for the surgeon. Complete rehabilitation of these patients is a team effort of specialists comprising of plastic surgeons and specialist dental surgeons. Successful management of such patients is a long exercise.

## CASE REPORT

An 18-year-old male patient presented to us with complaints of complete absence of teeth (anodontia), sparse hairs on scalp, absence of sweating and pigmentation of face. The patient also complained of heat intolerance, but was bothered mainly due to the absence of teeth. There was no similar history in the family. Past medical and treatment history was insignificant.

**Figure 1:**
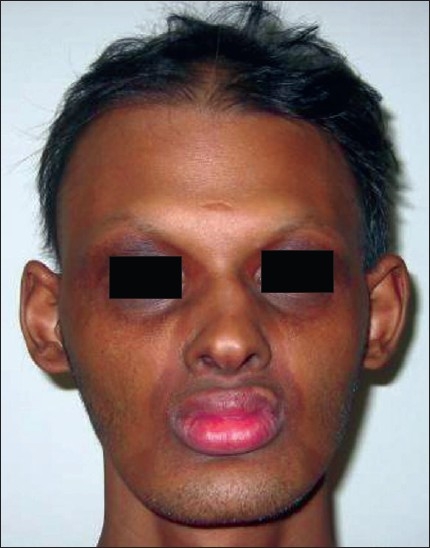
Pre-operative photograph of the patient showing the characteristic faecies

**Figure 2:**
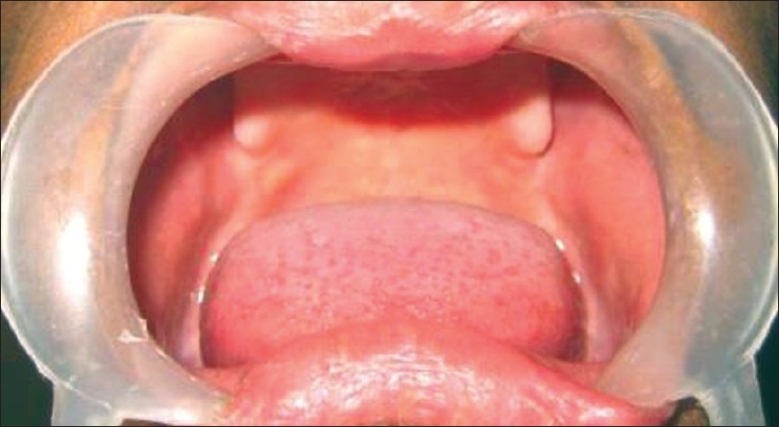
Pre-operative intraoral photograph showing absence of both upper and lower alveolus and complete anodontia

On examination, the patient had frontal bossing and sunken cheeks with thick everted lips [Figure 1]. Periorbital hyperpigmentation was present. Hair was found to be sparse and lustreless. The patient also had nail deformities with longitudinal ridges. The skin was dry and scaly. On intraoral examination, there was complete absence of teeth along with absence of both upper and lower alveolus [Figure 2]. Routine investigations were normal. OPG (Orthopantogram) was performed, which confirmed the absence of alveolus and teeth. Three-dimensional (3D) computed tomography scan of the facial skeleton was carried out for treatment planning. The patient was diagnosed as a case of hypohidrotic ectodermal dysplasia (Christ–Siemens–Tourine syndrome). Treatment was planned for maxillary and mandibular alveolar reconstruction with dental implants in multiple stages.

**Figure 3:**
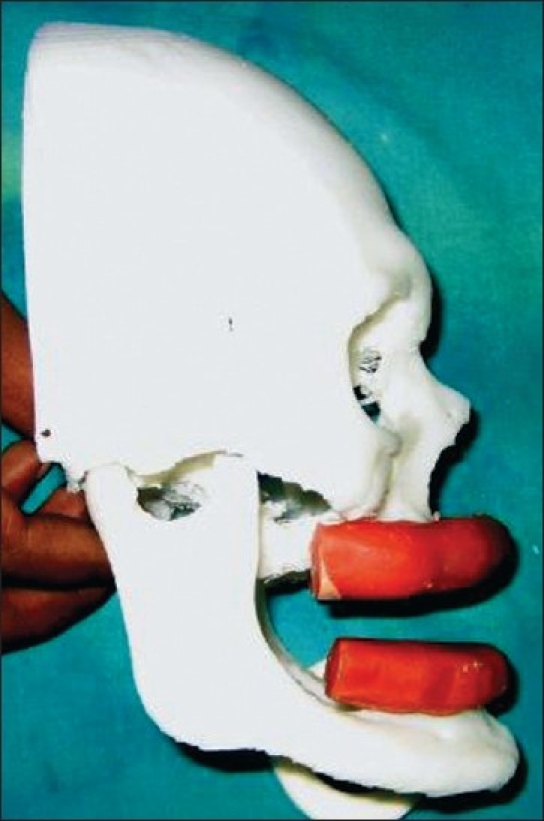
Three dimensional skull model of the patient used to assess the height and the width of the required upper and lower alveolus

In the first stage, upper alveolar reconstruction was planned using vascularised free fibula graft. 3D skull models with blocks of modeling wax were used to assess the height, width and length of the required alveolus [Figure 3]. Vascularised fibula flap was harvested from the right leg. Three osteotomies were performed to give it the required shape and length. It was put in place to reconstruct the upper alveolus and was covered with a split-thickness skin graft. Skin paddle was not used to avoid the bulkiness in the labial sulcus area. Skin graft was used over the muscle cuff surrounding the fibula.

The second stage was performed 6 months after the first surgery, when the previous flap was well settled. The following was done in this stage:

Reconstruction of the mandibular alveolus – This was planned and executed in a similar manner as the upper alveolus using the 3D skull models and the vascularised free fibula flap from the left leg.

Secondary ossseointegrated implants were inserted in the maxillary alveolus, which was reconstructed in the first stage. This was planned and executed by our prosthodontist and implantologist colleagues.

Primary ossseointegrated implants were inserted in the lower alveolus (fibular graft). This was also planned and executed by the prosthodontist and implantologists.

Wounds healed without any complications and third stage was planned after a further 6 months.

**Figure 4:**
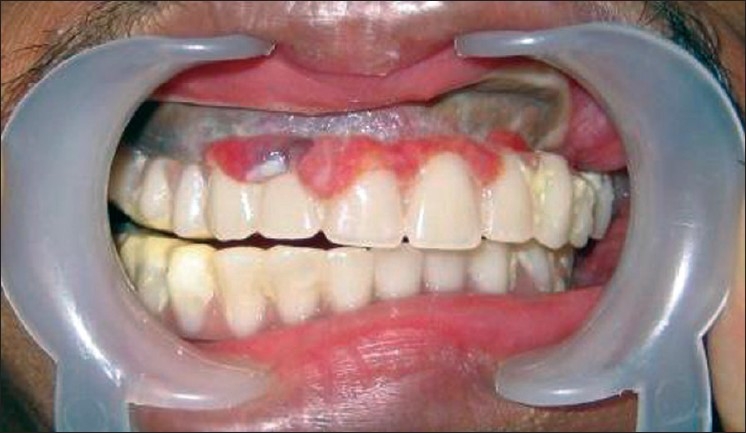
Postoperative intraoral photograph showing the overdentures

**Figure 5:**
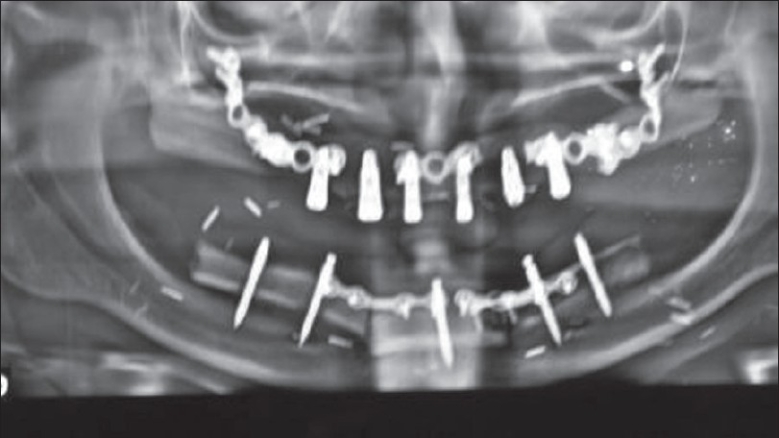
Postoperative OPG showing the reconstructed upper and lower alveolus (free fibula) and the dental implants

**Figure 6:**
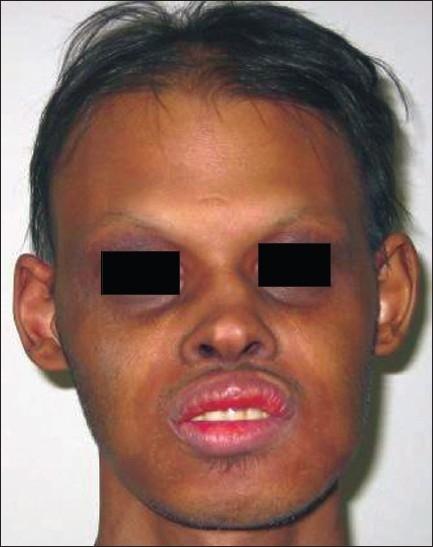
Postoperative photograph of the patient

In third stage, dental rehabilitation was carried out using overdentures for both maxilla and mandible [Figures 4 and 5]. The patient recovered well and is very happy, both functionally and aesthetically [Figures 4 and 6].

## DISCUSSION

The ectodermal dysplasias comprise a large, heterogenous group of inherited disorders that are defined by primary defects in the development of two or more tissues derived from the embryonic ectoderm. The tissues primarily involved are the skin, hair, nails, eccrine glands and teeth. The first published report of a patient with ectodermal dysplasia was in 1848 by Thurnam,[1] but the term "ectodermal dysplasia" was coined by Weech[2] in 1929. The condition is thought to occur in approximately one in every 100,000 live births.[3,4] Freire-Maia and Pinheiro[5] described 117 varieties of ectodermal dysplasia with multiple combinations of abnormal ectodermally derived structures. To date, more than 192 distinct disorders have been described.

The first classification system for ectodermal dysplasias was proposed by Freire-Maia and Pinheiro in 1982,[6] with additional updates in 1994 and 2001.[7,8] The patients were stratified into subgroups based on presence or absence of the following:

- Hair anomalies or trichodysplasias
- Dental abnormalities
- Nail abnormalities or onychodysplasias
- Eccrine gland dysfunction or dyshidrosis

Overall, the ectodermal dysplasias were classified into either group A disorders, which were manifested by defects in at least two of the four classic ectodermal structures as defined above, with or without other defects, and group B disorders, which were manifested by a defect in one classic ectodermal structure (1–4 from above) in combination with (5), a defect in one other ectodermal structure (i.e., ears, lips, dermatoglyphics). Eleven group A subgroups were defined, each with a distinct combination of two or more ectodermal defects (e.g., 2–4, 1–3, 1–4 from above). The group B disorders were indicated as 1–5, 2–5, 3–5 or 4–5 (from above).

With advances in the understanding of the genetic basis of the disease, newer classifications are proposed, which are based on the defects in cell–cell communication and signalling, adhesion, transcription regulation or development; e.g., the Priolo and Lagana classification in 2001 and the Lamartine classification in 2003.[9‐11] These classification systems are not of much relevance from the diagnosis and management point of view, as of today.

Of more relevance is the clinical classification. Clinically, hereditary ectodermal dysplasias may be divided into two broad categories:

1. X-linked hypohidrotic form (Christ–Siemens–Tourine syndrome), characterised by the classical triad of hypodontia, hypohidrosis and hypotrichosis and by characteristic dysmorphic facial features. Our patient was suffering from this syndrome, which presents with the following features:
  - The typical faecies, which is often not recognised until infancy, is characterized by frontal bossing, sunken cheeks, saddle nose, thick, everted lips, wrinkled, hyperpigmented periorbital skin and large, low-set ears.
  - Dental manifestations include conical or pegged teeth, hypodontia or complete anodontia and delayed eruption of permanent teeth.
  - Most patients have fine, sparse, lustreless, fair hair; therefore, little pigmentation in the hair shaft is observed microscopically and the medulla is often discontinuous. When medullation is present, a "bar code" appearance is often seen.
  - Onychodystrophy may occur, but is not common.
  - Extensive scaling of the skin and unexplained pyrexia secondary to anhidrosis may occur in the neonatal period. The development of a chronic eczematous dermatitis is common.
  - Other common signs are short stature, eye abnormalities, decreased tearing and photophobia.
2. Hidrotic form (Clouston's syndrome) that usually spares the sweat glands but affects the teeth, hair and nails. Most of the other clinical features are similar to that seen in the hypohydrotic form. It has an autosomal-dominant inheritance and is common in persons of French–Canadian ancestry.[12‐14]

Medical management is symptomatic. Patients with hypohidrosis require frequent consumption of cool liquids, wear cool clothing and should stay in a cool environment (air conditioning). Emollients for dry skin, artificial tears for patients with decreased lacrimation, saline sprays for nasal mucosa and antibiotics, if any infection occurs, may be required. Dental malalignment may require orthodontic treatment.

Surgery is required mainly for oral and dental rehabilitation. Cleft lip or palate, if present, require surgical repair. Other midfacial defects or hand/foot deformities may be corrected surgically.The oral rehabilitation of patients presenting with congenitally missing dentition is challenging because of the need for a multidisciplinary approach. Additional considerations, such as the patient's age, stage of growth, inherent anatomic deficiencies present in conjunction with the missing teeth, soft tissue defects, existence of malformed dentition, severe diastemas and psychological status, must be considered.[15] Patients with ectodermal dysplasia because of tooth absence have reduced alveolar bone with "knife-edge" morphology, making implant reconstruction a challenge. Therefore, patients frequently require bone grafting and sinus-lift procedures.[16‐18] In addition to bone deficiencies, soft tissue is absent, resulting in compromised aesthetics and presenting a risk of future biologic complications with the final restoration.[19,20] Our patient had absence of both upper and lower alveolus with anodontia along with soft tissue deficiency resulting in the typical faecies. The primary surgical considerations for placement of dental implants is the ridge width and a height that will suffice for placement of appropriately sized implants that will provide long-term functional oral rehabilitation success.[19]

Zygomaticus implants have been used to support a maxillary prosthesis,[21] but the results are not very encouraging.

Lypka *et al*. have reported use of the bilateral sinus-lift procedure to increase the height of the maxillary alveolus and cortical bone graft (from posterior ilium) to increase the width of the alveolus. It was followed by dental implants.[18]

The mandibular alveolar height can be increased using the 'tent-pole' technique as described by Marx *et al*.[22] The implants placed through a submental incision act as struts to support the overlying soft tissue to allow for bone graft healing. The disadvantages of this procedure are external scar and difficulty in placement of implants in the correct orientation.

Bone grafts are frequently used to create a base for the dental implants, as reported by many authors, e.g, Kearns *et al*.,[23] Shahrokhi *et al*.[24] and Fabio *et al*.[25] Most of those patients had partial alveolar atrophy and hence could be managed with small bone grafts. Our patient had complete atrophy of both the alveolus' hence requiring considerable length of bone for dental implants. 3D skull models, as used in our patient, help in planning height and width of the required alveolar reconstruction.Although free fibula flaps have been used in alveolar reconstruction for surgical defects following tumour excision, its use in patients of ED has never been reported.

We, with microvascular expertise, found free fibular flap the best alternative to reconstruct the alveolus. The results achieved are similar, if not better, compared with other reported methods, with fewer complications and morbidity.

Free fibula flap, used in our patient, provides both bony component and soft tissue component for alveolar reconstruction. Fibula has been proven to be one of the best bones when planning for osseointegrated implants, which can be performed primarily or secondarily.
